# The Clinical Society of Maryland

**Published:** 1899-06

**Authors:** 


### SOCIETY REPORTS.

### THE CLINICAL SOCIETY OF MARYLAND.

BALTIMORE, MD., March 17, 1898.

The meeting was called to order by the President, Dr. J. Williams Lord.

Drs. Paton and Rheuling gave a demonstration of serial sections of the brain.

"Report of a Successful Cæsarean Section." Dr. J. Whitridge Williams. This patient was a young white woman, aged 22, who had never been pregnant before and who first consulted Dr. Kirby last summer. At that time she stated that she had been troubled for several years with pain in the back and lower abdomen, and menstrual irregularity. The doctor examined her carefully and found a cystic tumor in the pelvis about the size of a cantaloup, connected he thought with the tube. He advised her to come back in the fall as it was not advisable to operate during the hot weather. At this second visit he found her pregnant, and some months later she fell into my hands. I found a well-nourished woman eight months pregnant, the fundus reaching up three or four fingers above the xiphoid cartilage, and the child presenting by the breech. The pelvic cavity was encroached upon by a tumor that extended almost entirely across its posterior part and filled out the curve of the sacrum. The antero-posterior dimension was reduced from 11cm. to about 7cm. or a little less. In other words, a very marked contraction of the pelvis. The tumor presented to the touch a doughy consistency and appeared to be under high pressure. We thought we had to deal with an ovarian cyst, and that the chances were it was dermoid in nature. I examined the woman the next day under chloroform and attempted to push the tumor up into the abdominal cavity, but found, though I introduced the whole hand, I could not budge the tumor, and concluded that some other means of delivery must be adopted. At that time a number of possibilities suggested themselves as the best means of treatment. In the first place had we seen the woman earlier we could have done a laparotomy, removed the tumor and allowed the pregnancy to go on the usual course, but we found the pelvis now so markedly encroached upon that normal delivery was impossible. The possibilities were these: Should we open the abdomen, remove the tumor, sew up and let labor go on? We felt that it was hardly fair to let the woman go into labor and try to express a full term child with an abdominal wall that presented a cicatrix only a few weeks old. A second proposition was to induce labor, but here we would labor under the disadvantage of leaving the tumor and delivering a child whose chances of living were not great. A third was to let the woman go to full term, puncture the tumor and then let delivery occur. We concluded, however, that the best and most conservative thing to do was a Cæsarean section, to take the living child out and then afterwards remove the tumor and thus relieve the woman of both at the same time.

I asked Dr. Kelly to see her with me, and he agreed with me that that was the best method. The woman acquiesced in the proposed operation. She stated that this pregnancy was the result of a single copulation, and accordingly we estimated the probable time of labor from the date of her last menstrual period and fixed our operation for one week previous to that time. We learned afterwards, however, that her statement was not correct, and she went into labor a week earlier than expected.

We operated on February 4, early in the morning. The woman fell into labor at 3:30 in the morning and was examined by Dr. Dobbin, who confirmed our previous opinion as to the position of the child. The cervix dilated to 4 or 5cm. in diameter, the membranes ruptured, and at 7 A.M., when I reached there, we prepared to do a laparotomy. The single examination made by Dr. Dobbin was the only one made during the last month of her pregnancy. She was given a bath, thoroughly cleansed, and placed upon the table in the horizontal position. I then made an incision in the median line of the abdomen 20cm. long. The woman was quite fat and there was some hemorrhage from the wound, which was readily controlled, however, by clamps. After we had opened the abdomen the uterus was directly under the wound, and we decided by the course of the round ligaments that the placenta was on the posterior wall of the uterus, so we made our incision in the anterior wall 15cm. long. As soon as we had cut through the wall I passed my hand in, seized the child by the leg, turned and extracted, and the entire time from the beginning of the operation until the child was in the hands of the nurse was something less than three minutes. I then seized the placenta, extracted that and began to close my uterine incision. The uterus was at no time taken from the abdominal cavity. The uterine incision was sewed up by a dozen or fifteen sutures, extending through the muscle to the deep decidua, but not through it. I think not more than four ounces of blood escaped from the woman during the operation, and the uterus contracted nicely to the size of two closed fists by the time the sewing was done.

We still had the tumor to deal with, and again passing my hand I brought up a cystic tumor from the right side; it was not such a cyst as we had expected, but a parovarian cyst filled with fluid. The abdomen was covered with sterile towels and the woman changed in position to that of Trendelenburg. The tumor was then removed in such a way as to leave the ovary on that side and part of the tube, simply removing the tumor. The abdominal wall was then closed with continuous catgut sutures, the muscle with silver wire, and the abdominal wall being quite fat we ran catgut through the subcutaneous tissue and closed the skin wound by subcutaneous silkworm sutures. A silver foil dressing was applied with the usual bandages. At the end of the operation the pulse was less rapid than when we began. As soon as the child was delivered the cord was clamped and cut and the infant handed to the nurse. It did not cry at first, but before she took it to the next room for the hot bath it began to cry, and so far as I know has been crying more or less satisfactorily ever since.

The entire operation took about fifty-three minutes, a great part of this being occupied in sewing up the uterus and abdominal walls. The child was in the nurse's hands within three minutes after the operation began. The woman made a most excellent recovery and left the hospital thirty-three days after the operation. The highest temperature at any time was 101, and that was only reached twice. The reason for the temperature I think can be found in the condition of the woman's breasts, as she did not wish to nurse the child and it was put in an institution. The breasts were strapped firmly, but in spite of the treatment they became tense and painful. However, I think she recovered as rapidly and with as little discomfort as do four women out of five with normal labors. The dressings were removed after eleven days and a single linear scar was all that was left to show that an operation had been done.

Now a few words on the subject of obstruction to labor by ovarian cysts. Of course a large number of women have ovarian tumors, and a large number of these have children without any particular difficulty. That is because the tumor rises up into the abdominal cavity as pregnancy advances; but if for any reason that tumor instead of rising up sinks down into the pelvis, it offers a very serious obstacle to labor. In 1849 an Englishman named Hart collected six cases of this kind, and in 1867 Dr. Playfair was able to collect reports of fifty-seven cases, which until recently represented the greatest aggregation of such cases known. In 1897, however, a physician of Aberdeen read a paper on this subject, and stated that he had been able to collect 126 other cases which, added to those of Playfair, make a total of 183, and from these he attempted to draw a number of important conclusions. Out of these 183 cases fairly accurate statements were made as to the nature of the tumor in 110 cases, and fifty per cent. of all were either dermoids or malignant tumors. This is of importance when we remember that the results are worse to the woman when the pelvis is impacted by a dermoid than when by an ordinary cyst, and statistics show that in fifty per cent. of the dermoid cyst cases women die in consequence. When we ask what was the direct effect of all the cysts upon the women we find that a little over thirty per cent. died either during labor or the weeks immediately following, and over half of all the children likewise died during the time of labor. These figures show then very clearly that an ovarian cyst impacted in the pelvis is a matter of grave importance and is a complication that may lead to the death of the woman in something like one-third of all the cases, even though properly attended to.

So we ask what is the best method to pursue when we meet with cases of this kind? I may say that the diagnosis is not usually made until the woman falls in labor, because while the woman may complain of some pain and discomfort, they usually pass unnoticed; in only about eighteen per cent. of the cases has the diagnosis been made previous to labor. If the diagnosis is made, however, what is the best method of treatment? I believe that if a cyst is diagnosed the woman should have an ovariotomy performed just as if she were not pregnant. On the other hand, if the diagnosis is not made until a late stage of the pregnancy I should say try to push the tumor up out of the pelvic cavity into the abdominal cavity; if necessary this should be done under anesthesia. Having so displaced the cyst we try to bring down one of the fetal poles and thus prevent the tumor falling into the pelvic cavity again. If we cannot do this we are brought face to face with the question whether we shall interfere at that time or allow the woman to go on to term before operating. I decided in my case on Cæsarean section at term, and I think the results show that I was justified.

In the vast majority of cases, as I have said, the diagnosis is not made until the time of labor. Of course we may try then to displace the cyst first, and if we succeed the head will engage spontaneously or by means of high forceps. This is not always a simple matter, however, for we may by pressure produce a necrosis which leads to infection of the cyst and future trouble. If we cannot push the tumor up, we find that all sorts of operative measures have been suggested. About eight different methods of treatment have been pursued and under each a certain number of women have fallen victims. A certain number of men leave these cases to nature, and as a result the women die. The most popular method of treatment perhaps has been to puncture the cyst through the vagina and then extract the child. Two other methods have been to attempt version or apply forceps and attempt to drag the child past the tumor. In a number of other cases craniotomy has been done and a dead child been delivered.

Others have performed a laparotomy for removal of the tumor and allow the labor to proceed naturally.

To consider all these methods I should say we should not leave the cases to nature, but make up our minds upon some method of treatment. As to the vaginal puncture, that would appear to be the most rational measure, and if we had known in our case in time the exact character of the cyst we might have done that, but the trouble is one cannot tell in many cases whether the cyst is parovarian, dermoid, or not, and the consequence is if we puncture we will in about fifty per cent. of the cases let the contents of the dermoid or a malignant tumor into the abdominal cavity and subject the woman to the chances of death from peritonitis. Again, the consequences of dragging the child out over an evacuated cyst is to subject the tumor mass to considerable pressure and the lowered vitality allows of infection from which the woman may die. Of course the application of forceps or any form of version in cases where the pelvis is obstructed is contra-indicated, unless the tumor is very small. Craniotomy is never indicated on a living child and to perform it on a dead child is only to subject the woman to more trouble. Laparotomy for removal of the tumor and spontaneous labor afterwards has been done several times, but has not proven very satisfactory. To subject a woman to laparotomy and immediately after that another operation is not a light matter, and I shall hesitate to adopt it. To remove the tumor by the vagina of course can be done in a certain number of cases, but we are operating in the dark, through tissues that may be easily infected.

The consequence is I should say that when we are called to see a woman in labor and find an obstructing tumor that offers serious obstacles to the passage of the child, if the woman be in good shape and competent assistance can be had, Cæsarean section in the hands of a competent man is the easiest and most conservative way of treating her. By all the other methods we deliver the woman by dangerous procedures and some weeks or months later have to do a laparotomy anyway to remove the tumor; so I think Cæsarean section is the proper operation under these circumstances. It may not always be practicable. The country physician may not be able to obtain proper assistance and may not be able to do the operation by himself. I confess I should not like to do it alone, and in such a case the best thing to do would be to puncture the tumor and extract the child, but in large cities where competent men can be called in I do not think that risk should be taken.

DISCUSSION.

Dr. B. B. Browne: This has been a very interesting talk, and the résumé of the subject has been extremely useful. I have never had any experience with ovarian tumors in pregnancy, but I have seen three cases complicated by fibroid tumors, and I think Dr. Williams has laid out the proper plan to be pursued in all these cases.

"Cerebellar Abscess of Otitic Origin," Dr. A. D. McConachie. The patient was a boy twelve years old, whose right ear had been discharging three years, following convalescence from typhoid fever; previous to that the boy was robust, always well and cheerful; since then he has been irritable, peevish and illy nourished. The usual measures for the arrest of the otorrhea were employed, with frequent cessation of discharge, to recur at intervals. About a year ago he had a marked recurrence of the otorrhea with certain cerebral manifestations, as vomiting, nausea and vertigo, followed by a cessation of the discharge, with coma, and as a consequence death was looked for. On chiseling into the mastoid it was found dense, the antrum being reached at half an inch depth and a small amount of cholesteatomatous material removed; the post-superior wall of the meatus was partially knocked down and a curette passed into the tympanic cavity. Free communication was established between the external meatus and antrum, manifested by syringing through the antrum into the tympanic cavity and out at the external meatus, and vice versa. On the tenth day after operation, Dr. McConachie found the boy semi-conscious, with marked retraction of the head, pupils dilated, eye ground normal, temperature normal, pulse 60, restless and apathetic. He advised further operative intervention, as he suspected either a cerebellar abscess or an extradural abscess, but was prevented from reaching the patient in time, and on the eleventh day after operation the boy died. At autopsy the meninges and sinuses were found normal, except a small area of meninges at the outer border of the right cerebellar lobe, where the meninges were necrotic, and about two ounces of pus escaped from a large pus cavity in the right lobe on removal of the brain from its cavity. The necrotic process had made an opening 2 mm. in diameter through the tympanic wall anterior to and above the lateral sinus.

The case is interesting in many particulars as not only indicating the possible menace to life which a neglected otorrhea entails, but also the symptomatic variability in cerebral complications of the same. A cerebellar abscess usually terminates in death when operative procedures are not used. The abscess contents escape and a new inflammatory action is set up. Abscesses have become encapsulated and remained quiescent for years without giving rise to serious trouble, but such cases are rare. The duty of the physician is to operate early if a successful result is to be hoped for, and the time to operate is when the differential diagnosis is made—a deep problem and sometimes very speculative. The complete removal of these obstructive conditions to respiration and proper ventilation of the tympanic cavity is often all the treatment that is necessary if the case is seen early, before any necrotic process has taken place in the tympanic or neighboring structure. When indicated the mastoid operation should be done, and done promptly, but other and more conservative measures should first be given a fair trial. There is a time, of course, in these cases when delay is dangerous and hesitancy may cause the life of the patient, and for these the radical surgical procedure is necessitated. These surgical measures may begin with the removal of adenoids or other obstruction to free respiration or tympanic damage; it may mean the incision of a drum at the right time for the removal of purulent contents; it may mean the removal of carious or necrotic ossicles, or tissue therein; it may mean the thorough intratympanic washing by antiseptics, or it may mean the opening of mastoid antrum and cells and the removal of other tissues, made necessary by involvement in the diseased process.

Dr. Reik: The case of Dr. McConachie's is an exceedingly interesting and a very important one, in that it illustrates so thoroughly the necessity for treatment in all cases of suppurative otitis media, and probably no surgeon in any branch of medicine would take the chances that the otologist takes with this class of patients. There has long been a popular impression that a long-continued discharge from the ear should not be stopped for fear of an eruption elsewhere on the body. It is needless to say that this is a surgical fallacy. Within very recent years the otologists have begun to apply surgical common sense to the treatment of these cases, and we cannot feel that a patient is safe until such discharge has been absolutely cured.

I wish to strongly emphasize the points Dr. McConachie has made with regard to the treatment—that it is wise first to try the simpler measures, such as local treatment by douching, etc., but if this is not successful surgical measures should be adopted. So long as there is a continuous discharge from the ear there is always danger of a cerebral abscess, and to wait until such symptoms develop renders the chances of recovery by operative procedure very much less favorable. The time to operate is before the abscess is started, or before general infection has taken place; in other words, to operate for the cure of the local suppurative trouble.

Dr. Reuling: The case referred to by Dr. McConachie does not seem to present any typical symptoms of brain abscess; but where the diagnosis is certain to any extent the opening of the brain is very advisable.

---

BALTIMORE, April 21st, 1899.

The meeting was called to order by the President, Dr. J. Williams Lord.

"Exhibition of Dermatological Cases," Dr. T. C. Gilchrist.

1st. *Lupus Erythematosus.* This patient has a typical distribution of the disease, assuming on the nose and both cheeks a butterfly appearance. It began on the cheek and then spread. The skin is thickened and covered with scales, which when removed show pedicles projecting from the under surface. The process of the growth is very slow.

This patient is perhaps even more typical than the other, for when the disease attacks the face it generally assumes the acute form. This patient has had the disease for about fifteen years. It began like scratch-marks on the side of the nose and then gradually spread over the cheeks. The pedicles under the scales dipped down into the sebaceous glands.

This disease was the chief subject of discussion at the last meeting of the American Dermatological Society and was also one of the important subjects considered at the British Medical Association last year. It is one of the most inveterate diseases of the skin and there are various opinions concerning its etiology. It was at first supposed to be of syphilitic origin and later that it was allied to tuberculosis of the skin, but that theory was abandoned after further investigation. Recently, however, this same theory has come up again, except that it is now believed to be due to a toxin of tuberculosis and not directly to the germ. The tuberculin test has been tried and the disease has yielded to the test, but that is not considered sufficient proof of a connection with tuberculosis, because other diseases of the skin which have no such connection will also yield to this test. That the lesion itself is tubercular is not admitted by dermatologists.

The treatment practically consists in simple mild remedies in the acute form and stronger ones for the chronic cases; that is, in the acute stage one may use an ointment of zinc oxide, but in the chronic cases perhaps the best results are obtained by an application of carbolic acid.

2. *Rodent ulcer.* This case began with a small scab on the left side of the forehead. This was followed by the appearance of a pimple, which after healing left a perfectly square scar. At the lower end of it is what appears to be a keloid. There was no history of injury and I was at first in doubt as to the diagnosis, but a section of the skin examined microscopically showed it to be a rodent ulcer which had been extremely slow in its growth. In the majority of cases these ulcers appear about the age of forty and treatment of course is total excision.

3. *Molluscum contagiosum.* So far as I know this disease has not been reported as having occurred in the colored race, but this is the third case we have seen. The rounded raised edge of the growth with a depression in the center is characteristic. Very often these molluscum growths spread all over the body, and I have had two or three medical men come to me who had contracted them from patients. The disease is extremely rare in this country.

Treatment consists in curetting out the bodies. The disease is probably of parasitic origin.

4. This patient has had an affection of the lips and mucous membrane of the mouth for twenty years, breaking out very severely about spring time every year. The affection was first described by Fordyce, of New York, as peculiar bodies, about pin-head size, buried in the lips and just at the inner side of the mouth. These bodies had never been described and after studying them he concluded that it was an affection of the epidermis. Montgomery, of San Francisco, later examined similar cases and found in the microscopic sections evidences of sebaceous glands. The affection then is similar to the presence of comedones on the face. I have excised from this patient a portion of the mucous membrane of the mouth and lip and one can see the sebaceous glands very well. The growths are due to choking up of the sebaceous secretion in the glands and their consequent enlargement. No name has been given to the affection as yet.

Adjournment.

---

### THE TREATMENT OF MALIGNANT PUSTULE.

Dr. Castroverde (*Revista de Medicina y Cirurgía prácticas*, December 15th) thus sums up his paper on this subject: 1. That the cauterization should be marginal if it is not to occasion much destruction and suffering, as it was practiced by the old-time surgeons. 2. The integrity of the escharotic crust must be preserved, using on it a protective cover to avoid contact with the infectious germs in the air. 3. Bichloride of mercury internally has given excellent results, as have also hypodermic injections of carbolic acid in two per cent. solution made twice or thrice daily in the thickness of the edematous zone and repeated until the edema has disappeared. The scar is removed by means of permanent irrigation of the pustule with carbolic acid, bichloride or bichromate solutions at a somewhat elevated temperature. By these means perfect antisepsis is maintained.

